# GFAP expression as an indicator of disease severity in mouse models of Alexander disease

**DOI:** 10.1042/AN20130003

**Published:** 2013-03-21

**Authors:** Paige L. Jany, Tracy L. Hagemann, Albee Messing

**Affiliations:** *Cellular and Molecular Pathology Training Program, University of Wisconsin-Madison, WI, U.S.A.; †Waisman Center, University of Wisconsin-Madison, WI, U.S.A.; ‡Waisman Center and Department of Comparative Biosciences, University of Wisconsin-Madison, WI, U.S.A.

**Keywords:** biomarker, CSF, ELISA, GFAP, luciferase, AxD, Alexander disease, BLD, biological limit of detection, CNS, central nervous system, COV, coefficient of variation, CSF, cerebrospinal fluid, GFAP, glial fibrillary acidic protein

## Abstract

AxD (Alexander disease) is a rare disorder caused by heterozygous mutations in GFAP (glial fibrillary acidic protein) resulting in accumulation of the GFAP protein and elevation of *Gfap* mRNA. To test whether GFAP itself can serve as a biomarker of disease status or progression, we investigated two independent measures of GFAP expression in AxD mouse models, one using a genetic reporter of promoter activity and the other quantifying GFAP protein directly in a manner that could also be employed in human studies. Using a transgenic reporter line that expresses firefly luciferase under the control of the murine *Gfap* promoter (*Gfap*-*luc*), we found that luciferase activity reflected the regional CNS (central nervous system) variability of *Gfap* mRNA in *Gfap^+/+^* mice, and increased in mice containing a point mutation in *Gfap* that mimics a common human mutation in AxD (R239H in the human sequence, and R236H in the murine sequence). In a second set of studies, we quantified GFAP protein in CSF (cerebrospinal fluid) taken from three different AxD mouse models and littermate controls. GFAP levels in CSF were increased in all three AxD models, in a manner corresponding to the concentrations of GFAP in brain. These studies demonstrate that transactivation of the *Gfap* promoter is an early and sustained indicator of the disease process in the mouse. Furthermore, GFAP in CSF serves as a potential biomarker that is comparable between mouse models and human patients.

## INTRODUCTION

AxD (Alexander disease) is a rare neurodegenerative disorder caused by heterozygous mutations in the gene encoding GFAP (glial fibrillary acidic protein), the major intermediate filament protein of astrocytes (Brenner et al., 2001). The mutations appear to act in a gain-of-function fashion, with a rise in GFAP levels above a toxic threshold serving as a key step in pathogenesis (Brenner et al., 2009). However, the mechanisms underlying the accumulation of GFAP are not completely understood. Studies from cell culture models implicate impaired degradation through the proteasomal pathway as one cause of GFAP increase, despite a near simultaneous increase in autophagy (Tang et al., 2006, 2008). In addition, analysis of both human tissues and mouse models reveal increases in GFAP mRNA (Hagemann et al., 2005, 2006), suggesting that increased synthesis could also contribute to an accumulation of GFAP protein. Given the very slow turnover rate for GFAP protein (Chiu and Goldman, 1984; Morrison et al., 1985; DeArmond et al., 1986; Price et al., 2010), any changes in GFAP promoter activity and increased synthesis would have long-lasting effects. GFAP accumulation may also exacerbate pathology through the formation of positive feedback loops that impact both synthesis and degradation (Messing et al., 2012).

Regulation of GFAP expression occurs primarily at the level of GFAP transcription, rather than through alterations in mRNA stability or translational efficiency (Brenner, 1994). Whether the changes in GFAP mRNA noted above reflect feedback stimulation of *Gfap* promoter activity is not known. Methods for investigating promoter activity *in vivo* have been greatly facilitated by the development of transgenic models that carry reporter genes under the control of cell-specific regulatory elements (Cui et al., 1994). One such reporter is the *Gfap-luc* mouse, which expresses the firefly luciferase gene under the control of a 12 kb 5′ flanking region from the murine *Gfap* gene (Zhu et al., 2004). These mice have previously been shown to exhibit increases in luciferase activity in response to a variety of insults or diseases that also result in astrogliosis and raised levels of GFAP mRNA, including kainic acid-induced seizures (Zhu et al., 2004), bacterial infection (Kadurugamuwa et al., 2005), inflammation (Luo et al., 2008), stroke (Cordeau et al., 2008), scrapie (Tamgüney et al., 2009), motor neuron degeneration (Keller et al., 2009) and expression of mutant APP (amyloid precursor protein) (Watts et al., 2011).

With the goal of identifying indicators of disease severity and progression that could be easily monitored in mouse models of AxD, we sought to test whether the *Gfap-luc* reporter is responsive to the novel genetic injury represented by the expression of mutant GFAP. We also tested the hypothesis that the elevations in GFAP protein previously detected in brain parenchyma are also reflected at the level of CSF (cerebrospinal fluid), a site that is readily amenable to biopsy in human patients and in which increased GFAP has been observed in AxD patients (Kyllerman et al., 2005). We found distinct variations in GFAP expression and response to AxD mutations in different brain regions of the mouse models. Increased activity from the *Gfap-luc* reporter is evident at the whole brain level as soon as 14 days after birth and this increase is sustained through at least 6 months of age. We also found that GFAP is detectable at low levels in the CSF of control mice, but is elevated in three different AxD models to degrees corresponding to the amount of GFAP accumulation in brain.

## MATERIALS AND METHODS

### Mice

The experiments described here were approved by the Animal Care and Use Committee for the Graduate School at the University of Wisconsin, Madison. The *Gfap^R236H/+^* and *Gfap^R76H/+^* lines of mice contain knock-in mutations at the endogenous *Gfap* locus that are homologous to common human AxD-associated mutations (R239H and R79H, respectively) (Hagemann et al., 2006). The *GFAP^Tg^* mice are a transgenic line (Tg73.7) that overexpresses wild-type human GFAP (Messing et al., 1998). The *Gfap*-*luc* mice are a transgenic line that expresses firefly luciferase under the control of a 12 kb murine *Gfap* promoter (Zhu et al., 2004). *Gfap^tm1Mes^* mice carry a null mutation at the *Gfap* locus (McCall et al., 1996), and were used as negative controls in the validation of the GFAP ELISA (see below). All mice were maintained in the FVB/N background and were housed under a 14–10 light–dark cycle with *ad libitum* access to food. Samples were collected from mice at 8 weeks of age. Brains were either divided sagittally into two equal halves, or dissected into individual regions [olfactory bulb, frontal cortex (anterior to Bregma, dorsal grey matter containing little or no white matter), hippocampus, cerebellum and brain stem] along with cervical spinal cord. Tissues were immediately frozen in liquid nitrogen and stored at −80°C until further processing.

### Quantification of *Gfap* promoter activity

*Gfap*-*luc* transgenic mice were used as indirect reporters of *Gfap* promoter activity. Firefly luciferase activity was quantified using the Dual-Glo luciferase kit (Promega), according to the manufacturer's directions. Briefly, tissues were homogenized in reporter lysis buffer (125 mg tissue per ml buffer), centrifuged at 17500 ***g*** for 20 min at 4°C, and the supernatant taken for analysis. An aliquot of this supernatant (40 μl for half brains, 20 μl for smaller pieces) was diluted 1:1 in firefly luciferase reagent and allowed it to incubate at room temperature (22–24 °C) for 12 min. The signal intensity was then determined with a GloRunner Microplate Luminometer (Turner Biosystems). A separate aliquot was assayed for total protein using a BCA Protein Assay kit with BSA as a standard (Thermo Scientific). Values are expressed in arbitrary luminescent units per mg protein.

### Quantification of *Gfap* mRNA

Tissues were homogenized in TRIzol Reagent (Invitrogen) according to the manufacturer's directions. cDNA was synthesized using Superscript III (Invitrogen) according to the manufacturer's protocol. Specific transcripts were then quantified by real-time PCR using Power SYBR Green Master Mix and an Applied Biosystem 7500 Real-Time PCR system as described previously (Hagemann et al., 2005). Primer sets were the following: *Gfap* (forward: 5′-CAACGTTAAGCTAGCCCTGGACAT-3′, reverse: 5′-CTCACCATCCCGCATCTCCACAGT-3′), 18S ribosomal RNA (forward: 5′-CGCCGCTAGAGGTGAAATTCT-3′, reverse: 5′-CGAACCTCCGACTTTCGTTCT-3′) and TATA-box-binding protein (forward 5′-GCACAGGAGCCAAGAGTGA-3′, reverse 5′-CCCACCATGTTCTGGATCTT-3′). *Gfap* transcript levels were normalized to both the 18S ribosomal and TATA-box-binding protein RNAs. Both normalizations yielded similar results. Data shown were normalized to the 18S ribosomal RNA.

### Collection of CSF

CSF was collected from mice according to the method of DeMattos et al. (2002). Mice were anaesthetized with avertin (400–600 mg/kg intraperitoneally). A midline sagittal incision was made over the dorsal aspect of the hindbrain and three muscle layers carefully peeled back to expose the cisterna magna. The membrane covering the cisterna magna was pierced with a 30 gauge needle, and CSF was collected immediately using a flexible plastic pipette. Approximately 10 μl of CSF was collected per animal, and stored at −80°C until further processing.

### Quantification of GFAP protein

Brain samples were homogenized (w/v, 1:16) in 2% SDS, 50mM Tris/HCl (pH 7.4), 5mM EDTA (pH 7.4), 1mM PefablocSC (Sigma-Aldrich) and 1× Complete Proteinase Inhibitor (Roche) using a Geno/Grinder tissue homogenizer (SPEX CertiPrep). After homogenization, samples were boiled for 15 min. Samples were then diluted in PBS (Fisher, #BP399-4) and protein concentration determined using a BCA Protein Assay kit with BSA as a standard (Pierce). This same diluted sample was then used for the GFAP ELISA as described below.

GFAP protein was quantified using a sandwich ELISA as previously described (Petzold et al., 2004; Hagemann et al., 2009), with minor modifications. Briefly, a microtitre plate (Nunc MaxiSorp) was coated with a cocktail of monoclonal antibodies (Covance, SMI-26R) diluted in PBS (Fisher). Plates were blocked with 5% (w/v) non-fat dried skimmed milk powder in PBS before addition of samples or standards diluted in PBS with 0.05% (v/v) Tween 20 and 1% (w/v) BSA (Sigma, #A7030). Antibody incubation steps were performed in 5% (w/v) non-fat dried skimmed milk powder–PBS, and washing steps were performed in PBS–Tween 20 (without BSA). Standard curves were generated using bovine GFAP (Fitzgerald Industries International, # RDI-PRO62007) diluted in PBS–1% BSA. Assay volumes consisted of 100 μl per well when analysing brain samples or 50 μl per well when analysing CSF. Samples were diluted with PBS–Tween 20–BSA as needed to bring their GFAP values within the linear range of the standard curve.

In the case of CSF samples from controls, which typically had low levels of GFAP, the dilutions required to bring the reaction volume up to the minimum 100 μl for duplicate wells often meant that the values fell below the BLD (biological limit of detection) (see below). We therefore used pooled samples for controls, replicated as four independent sets derived from 3–4 animals per group, and diluted a minimal amount (36 μl CSF+64 μl ELISA buffer). Mice from the AxD models had high-enough levels of GFAP to allow dilutions from individual samples, typically 1:16–1:120. After washing, a rabbit polyclonal antibody (Dako, # Z334) was used to detect the GFAP followed by a peroxidase conjugated anti-rabbit IgG secondary antibody (Sigma, # A6154). The peroxidase activity was detected with SuperSignal ELISA Pico Chemiluminescent Substrate (Pierce, Thermo Scientific) and quantified with a GloRunner Microplate Luminometer (Turner Biosystems). GFAP values for the brain samples were expressed as per mg total protein and for the CSF samples as ng/l.

Under these conditions the lower limit of detection (defined as three S.D. above the mean of replicate blank samples) was 11 ng/l, and the BLD (defined as the lower limit of detection plus three S.D. of a known low-concentration sample) (Westgard, 2008) was 21 ng/l for a 50 μl reaction volume. The intra-assay COV (coefficient of variation), determined using the bovine GFAP standard at 33 ng/l in ten sets of triplicate wells, was 13%. The inter-assay COV, determined from the pooled CSF samples taken from *GFAP^Tg^* mice (with values higher on the standard curve), divided into multiple identical aliquots to allow ten independent assays on different days, was 11%. Samples from *Gfap*-null mice give readings that are below the BLD in this assay (results not shown), thus validating its specificity.

### Statistical analysis

For the comparisons of *Gfap* promoter activity, mRNA and protein shown in Figure 2, data were first log transformed [*Y*=log(*Y*)] to equalize the S.D. (the Figure shows the original untransformed data) and then analysed using a two-way ANOVA with *post-hoc* Bonferroni-corrected *t*-tests. All other data analyses used a one-way ANOVA with *post-hoc* Bonferroni-corrected *t*-tests between *Gfap^R236H/+^* and *Gfap^+/+^*. The error shown for the fold-changes between *Gfap^R236H/+^* and *Gfap^+/+^* mice in Figure 2 is the calculated propagated error for each data point. The CSF analysis also required log transformation of the data to equalize the S.D., and was then analysed using a one-way ANOVA with *post-hoc* Bonferroni-corrected *t*-tests.

## RESULTS

### *Gfap* promoter activity, mRNA and protein vary by brain regions in *Gfap^+/+^* mice

**Figure 1:**
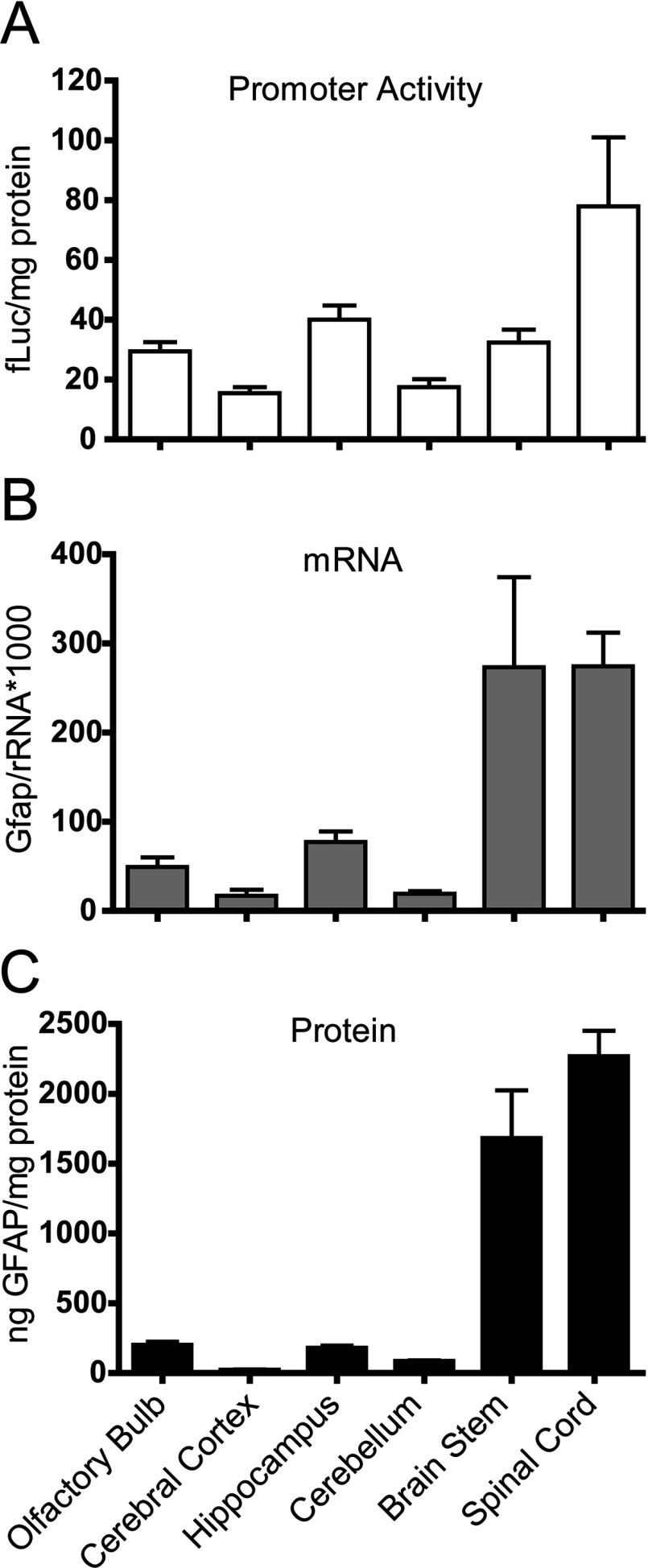
*Gfap* promoter activity, mRNA and protein in *Gfap^+/+^* mice *Gfap* promoter activity (**A**), mRNA (**B**) and protein (**C**) concentrations vary among regions, with the highest levels in spinal cord for all three measures of *Gfap*. Presented as means±1 S.D. (*n*=3–6, males). fLuc, firefly luciferase.

We sought to test whether *Gfap-luc* transgenic reporter mice, which express the firefly luciferase under the control of the murine *Gfap* promoter, thus serving as an indirect monitor of promoter activity, faithfully reflect the regional variation in GFAP expression that is known to exist in the rodent CNS (central nervous system). In *Gfap*-*luc* transgenics that are wild-type at the *Gfap* locus (*Gfap^+/+^*), luciferase activity was highest in the spinal cord, intermediate in olfactory bulb, hippocampus and brain stem, and lowest in cerebral cortex and cerebellum (Figure 1A). For comparison, we measured *Gfap* mRNA levels in these same regions, and found that spinal cord and brain stem had the highest levels, and cerebellum and cerebral cortex the lowest (Figure 1B). Total GFAP protein levels, as measured by ELISA, followed essentially the same pattern as mRNA (Figure 1C). Hence, luciferase activity from the reporter mice mirrored relative mRNA and protein levels for all regions that were examined, with the exception of brain stem.

### Regional *Gfap* gene activity in *Gfap^R236H/+^* mice

**Figure 2:**
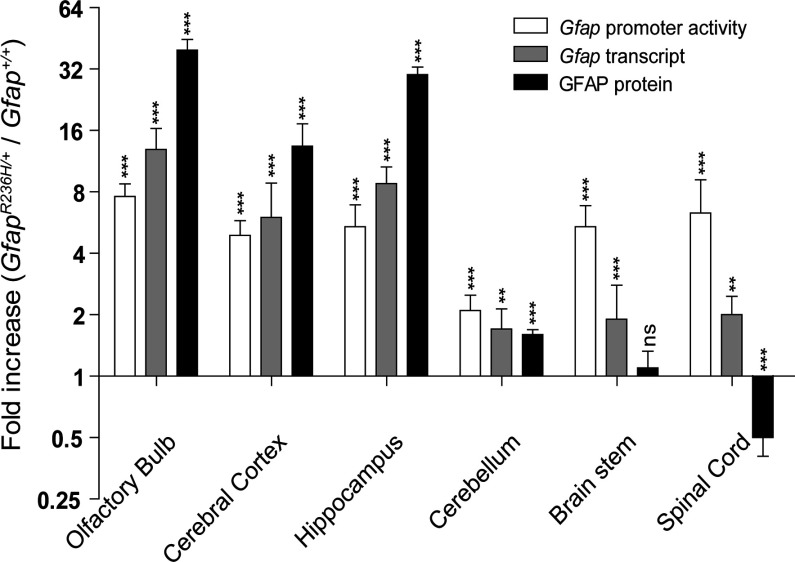
Levels of *Gfap* promoter activity, mRNA and protein in *Gfap^R236H/+^* mice compared with *Gfap^+/+^* mice A comparison of the fold changes seen in *Gfap* promoter activity, mRNA and protein shows that all three are reflective of the other except in spinal cord and brain stem (two-way ANOVA with *post-hoc* Bonferroni *t*-test; ns, not significant; **P<0.01;*** *P*<0.001 compared with wild-type). Presented as an average fold-change ±1 S.D. on log 2 scale (*n*=3–6 per genotype, males).

Mice carrying point mutations at the *Gfap* locus mimicking those found in AxD patients have elevated levels of *Gfap* mRNA and protein (Hagemann et al., 2006; LaPash Daniels et al., 2012), although not uniformly in all regions of the CNS. In order to test whether these changes in GFAP expression are mirrored by the *Gfap-luc* reporter, we crossed the reporter with a knock-in of the R236H mutation (*Gfap^R236H/+^*), and examined all three measures of GFAP expression in various CNS regions of 8-week-old mice (Figure 2).

*Gfap* promoter activity significantly increased in all six regions of *Gfap*-*luc*;*Gfap^R236H/+^* mice compared with *Gfap*-*luc*;*Gfap^+/+^* littermates. The largest fold-increase of *Gfap* promoter activity was in the olfactory bulb (7.6±1.2), and the smallest in cerebellum (2.1±0.4), with intermediate changes for the other regions. Quantification of *Gfap* mRNA levels in the same mice revealed fold changes similar to those found for promoter activity in olfactory bulb, hippocampus, cerebral cortex and cerebellum, and more modest increases in brain stem and spinal cord.

We then measured total GFAP protein levels in the same brain regions from *Gfap* mutant compared with wild-type mice. As previously described (Hagemann et al., 2006; LaPash Daniels et al., 2012), both olfactory bulb and hippocampus displayed substantially increased levels of GFAP (40-fold ±5 for olfactory bulb; 30±3 for hippocampus), but well beyond the fold changes observed for promoter activity and mRNA. Cerebellar GFAP increased by a modest amount, commensurate with the changes in promoter activity and mRNA. Interestingly, the two regions of the CNS with the highest basal levels of GFAP protein in wild-type mice, brain stem and spinal cord (see Figure 1C), displayed marked discrepancies between promoter activity/mRNA levels and protein levels when evaluated in the *Gfap^R236H/+^* mutants. Although promoter activity increased about 5-fold in both the brain stem and the spinal cord, mRNA levels increased by a lesser amount, and protein remained unchanged in brain stem and actually decreased in spinal cord. Hence, when examined in the context of the injury response associated with expression of mutant GFAP, the precise relationships between promoter activity, transcription and protein accumulation is complex and varies considerably between different regions of the CNS.

### Induction of *Gfap* promoter and increase in mRNA is not affected by gender

**Figure 3:**
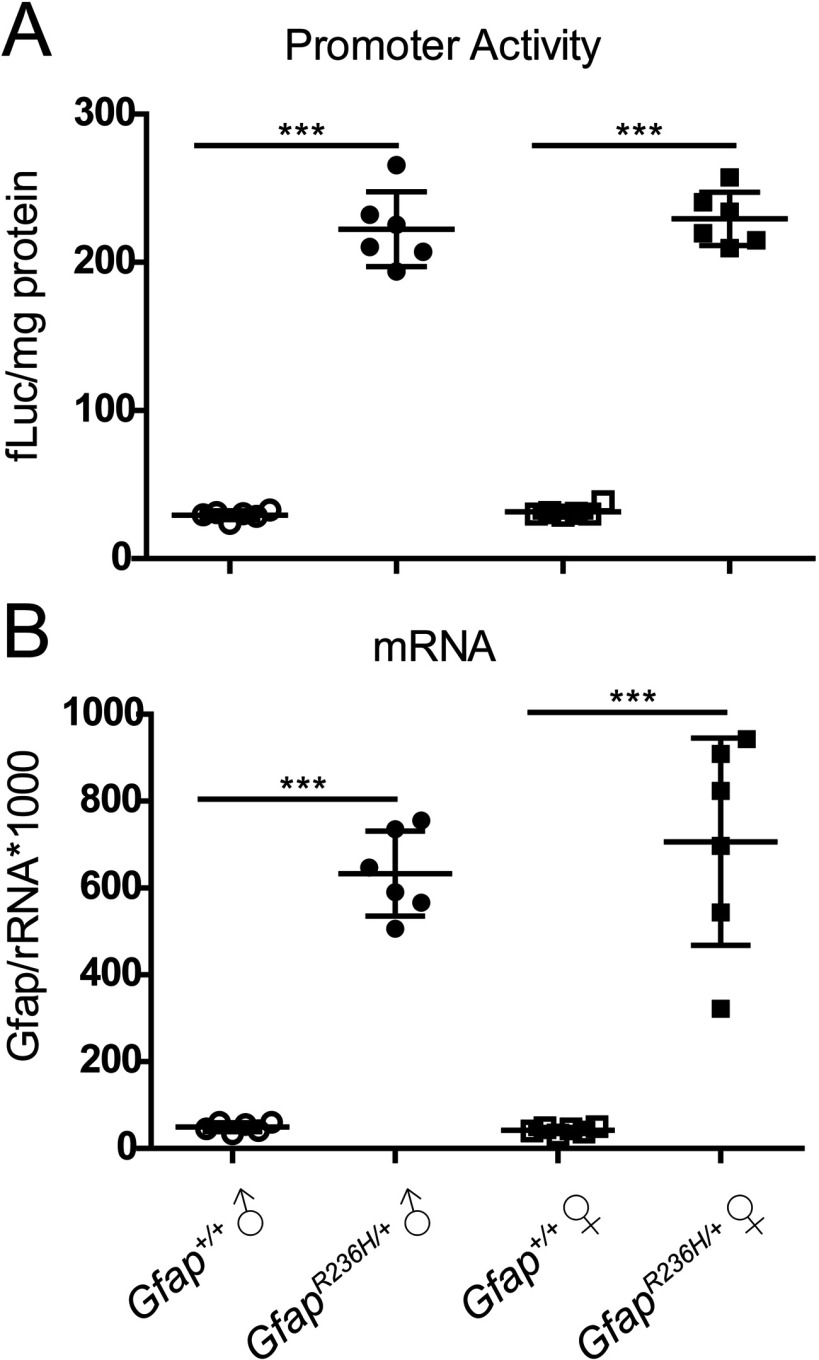
*Gfap* promoter and mRNA levels by gender While *Gfap* promoter activity and mRNA are elevated in *Gfap^R236H/+^* mice compared with *Gfap^+/+^*, the effect is similar in males and females in olfactory bulb (illustrated) as well as in other brain regions (results not shown). (One-way ANOVA with *post-hoc* Bonferroni *t*-test; *** *P*<0.001). Presented as means±1 S.D. (*n*=6 male and 6 female mice per genotype).

Previous studies have implicated oestrogens in the hormonal regulation of GFAP expression (Laping et al., 1994; Stone et al., 1998; Levin-Allerhand et al., 2001; McAsey et al., 2006; Cho et al., 2010). We therefore tested whether the changes in *Gfap* promoter activity and mRNA observed in the context of the R236H point mutation were influenced by gender. However, none of the regions examined showed any differences between males and females [olfactory bulb illustrated in Figure 3, other regions (results not shown)].

### Time course of induction of *Gfap* promoter activity in *Gfap^+/+^ and Gfap^R236H/+^* mice

**Figure 4:**
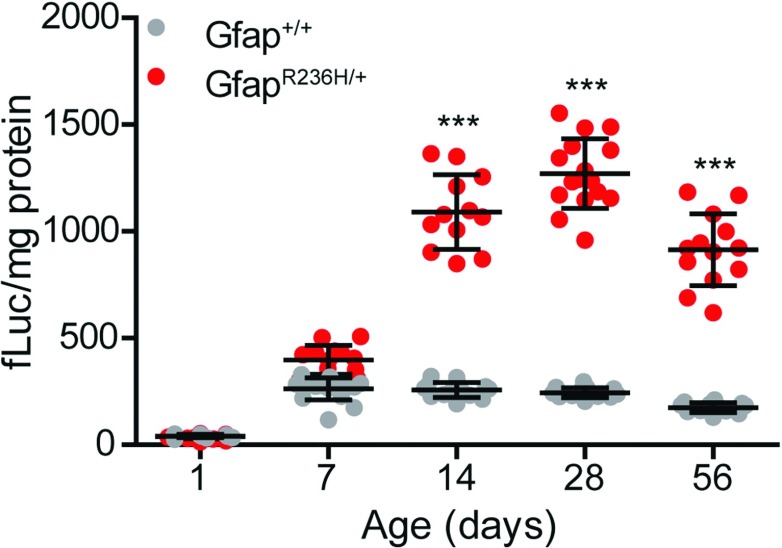
Developmental time course of *Gfap* promoter activity in *Gfap^R236H/+^* brain *Gfap* promoter activity is significantly elevated in *Gfap^R236H/+^* mice over *Gfap^+/+^* controls as early as postnatal day 14 (p14) and remains elevated through 8 weeks of age (p56). (One-way ANOVA with *post-hoc* Bonferroni *t*-test; *** *P*<0.001). Presented as means±1 S.D. (*n*=12–25).

To determine when *Gfap* promoter activity becomes elevated in *Gfap* mutant mice during development, we analysed luciferase activity in whole brains (including olfactory bulbs) from *Gfap*-*luc*;*Gfap^R236H/+^* mice compared with *Gfap*-*luc* littermate controls at various ages beginning at post-natal day 1 (p1). Luciferase activity displayed about a 4-fold increase in *Gfap^R236H/+^* mice as early as p14, and remained significantly elevated through 8 weeks of age (Figure 4). Using a different luciferase assay, we examined mice at 6 months of age, and found that elevated promoter activity was still evident (results not shown).

### GFAP in CSF of *Gfap* mutant and *GFAP* transgenic mice

**Figure 5:**
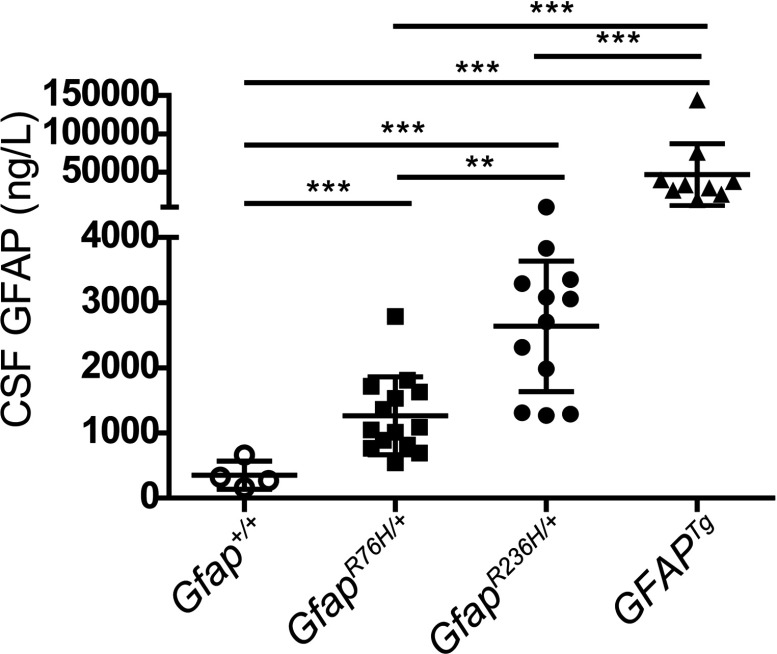
GFAP protein in CSF of mouse models of AxD GFAP is elevated in CSF of all three mouse models of AxD (*Gfap^R76H/+^*, *Gfap^R236H/+^* and *GFAP^Tg^*) compared with *Gfap^+/+^* controls. Presented as means±1 S.D. on a split linear scale (*n*=9–14, males). (One-way ANOVA with *post-hoc* Bonferroni *t*-test; **P<0.01; ***P<0.001).

In pilot studies of CSF from individual control mice using standard dilutions required to reach minimal assay reaction volumes (see the Materials and Methods section), one-third of the samples yielded values that were below the BLD of the assay. To permit an accurate determination of the values in controls, we therefore created four separate sets of pooled samples, each composed of CSF taken from 3–4 *Gfap ^+/+^* mice. In this manner, the volume of CSF was sufficient for use with minimal dilution in the GFAP ELISA, thus increasing the sensitivity for detection. We then measured GFAP in CSF from individual mice of three AxD models that have previously been found to have varying levels of GFAP accumulation in brain parenchyma: the *Gfap^R236H/+^* mice described above, the *Gfap^R76H/+^* knock-in model (homologous to the human R79H mutation and intermediate in levels of GFAP between control and *Gfap^R236H/+^* mice), and the transgenic that overexpresses human wild-type GFAP to very high levels (Hagemann et al., 2006). We found that GFAP in the CSF of *Gfap^+/+^* mice was detectable in all four pooled samples (354±217 ng/l, means±S.D.), whereas GFAP in the CSF of all three AxD models was significantly elevated compared with controls, and in the same rank order as expected from brain (1264±596 for *Gfap^R76H/+^*; 2637±1001 for *Gfap^R236H/+^*; 46676±40533 for *GFAP^Tg^* mice; *n*=9–14 in each group). In addition, the differences in CSF levels between the AxD model groups were statistically significant in all pair-wise comparisons (Figure 5).

Brain levels of GFAP were then determined in each individual from which the CSF samples were taken in the three AxD models. Although the between-group comparisons showed distinct differences, within each group the correlation between CSF and brain levels for individuals was not significant (*Gfap^R76H/+^*, *r*^2^=0.27; *Gfap^R236H/+^*, *r*^2^=0.38; *GFAP^Tg^*, *r*^2^=0.07; analysed by the Spearman non-parametric correlation) (Supplementary Figure S1 at http://www.asnneuro.org/an/005/an005e109add.htm).

## DISCUSSION

While mutations in *GFAP* are clearly the initiating event in AxD, ultimately the total amount of GFAP rises with secondary consequences for a multitude of astrocyte functions. What accounts for this increase is not clearly understood, although changes in the rates of both degradation and synthesis have been proposed (Messing and Brenner, 2013). Whether documented elevations in GFAP mRNA can be accounted for by increased activity of the *GFAP* promoter has not previously been addressed, and whether the putative changes in promoter activity and increases in protein can be exploited for drug discovery and clinical studies is also not known. Here we demonstrate, using several mouse models of AxD, that the activity of the murine *Gfap* promoter is indeed increased as an early and sustained response following the expression of mutant GFAP *in vivo*. Furthermore, we find that the levels of GFAP in CSF increase, roughly in parallel with the degree of increase present in brain. These results provide a foundation on which to build future studies seeking to interfere with the toxic increase in GFAP as a therapeutic goal (Messing et al., 2010).

Activation of the *GFAP* promoter with resulting increase in synthesis is considered a fundamental property of reactive astrocytes. Although the precise mechanisms mediating this increase are still topics of active investigation, numerous studies suggest that at least some of the regulatory elements for the reactive response lie within a 2.2 kb fragment of the 5′ region of the gene. Previous studies from our laboratory utilized this 2.2 kb human *GFAP* promoter construct to create dual luciferase reporter mice for monitoring of *GFAP* promoter activity, and in crosses of these luciferase reporters with the *Gfap^R236H/+^* mutants we found only a modest increase in luciferase activity, which disappeared by 8 weeks of age (Cho et al., 2009). In contrast, the *Gfap*-*luc* mouse utilized in the present study, which differs from our dual luciferase mouse in several respects, reports a much larger and sustained increase in promoter activity. In addition, the fold-increase in luciferase activity more closely resembles the fold change in mRNA levels, suggesting as well that the predominant regulation of GFAP expression occurs at the level of transcriptional activation.

The degree to which elevated levels of GFAP protein *per se* provide the initial stimulus leading to promoter activation is not yet clear. Future studies will examine the response of the *Gfap*-luc reporter in transgenic lines that overexpress wild-type rather than mutant GFAP, although it will be important to closely match the levels of GFAP protein between lines. It is possible that mutant GFAP acts in essentially the same manner as wild-type, but more efficiently and at lower levels of overexpression.

That expression of GFAP varies considerably in different regions of the CNS and in different populations of astrocytes is well known. For instance, both mouse and human data reveal the highest levels of both mRNA and protein in spinal cord and brain stem, followed in descending order by hippocampus, olfactory bulb, cerebellum and cerebral cortex (Palfreyman et al., 1979; Chen et al., 1993; Martin and O’Callaghan, 1995; Lein et al., 2007). In contrast, one study of rats found cerebellum to be higher than hippocampus (Martin and O’Callaghan, 1995). The reasons for these differences are not clear, but could reflect variations in either the number or composition of astrocyte sub-types among regions. Recent studies of human astrocytes differentiated *in vitro* from embryonic stem cells reveal that those induced to a ‘caudalized’ phenotype through exposure to retinoic acid express higher levels of GFAP than those following the default rostral pathway of differentiation (Krencik et al., 2011). Our studies support the idea that regulation of GFAP levels occurs primarily at the level of transcription, as originally proposed by Brenner (1994), with a notable exception being spinal cord of the GFAP mutants. It is interesting that while the type I form of AxD is characterized by predominance of forebrain lesions, the type II form shifts to a hindbrain distribution, with many adult-onset patients experiencing atrophy of the medulla and cervical spinal cord (Prust et al., 2011; Messing et al., 2012).

Why GFAP should appear in the CSF is not at all clear. Studies in a wide variety of human disorders document elevations of GFAP that are typically attributed to cell death, although most of these studies also find detectable levels even in healthy controls (Liem and Messing, 2009). One possibility is that GFAP, despite being a cytoskeletal protein, is normally secreted from astrocytes, as has been found to occur for vimentin from macrophages (Mor-Vaknin et al., 2003). Studies of the astrocyte secretome have largely utilized cell cultures and are complicated by the problem of correcting for contamination by cytoplasmic contents – most reports finding that vimentin and not GFAP is secreted (Delcourt et al., 2005; Keene et al., 2009; Greco et al., 2010). Dowell et al. (2009) did find GFAP in the supernatant from cultured mouse astrocytes, but considered it a contaminant. Moore et al. (2009), in a study using rat astrocyte cultures, found neither vimentin nor GFAP. Whether the GFAP mutations and brain overexpression that have been found in AxD lead to astrocyte death, with consequent release of cytoplasmic contents into the extracellular space and eventually CSF, is also not known. Evidence for an increase in cell death following expression of mutant GFAP has been obtained from cell culture models (Mignot et al., 2007; Cho and Messing, 2009), but no similar data have been obtained from the *in vivo* models of disease (Hagemann et al., 2006; Tanaka et al., 2007). The amount of GFAP present in the CSF of *GFAP^Tg^* mice is sufficiently high as to be detectable by mass spectrometry, thus opening new possibilities for methods of quantification and characterization (Cunningham et al., 2013).

A major problem facing therapeutic research is the identification of suitable biomarkers that reflect key pathways of disease and could be responsive to drugs or other types of treatment. The response of the *Gfap-luc* reporter mouse clearly demonstrates transactivation of the *Gfap* promoter, perhaps as a final common pathway in the putative positive feedback loops that lead to toxic accumulation of GFAP (Messing et al., 2012). Activation of the *Gfap* promoter may also represent a step in pathogenesis that is amenable to drug discovery or screening efforts (Cho et al., 2010; Messing et al., 2010). In addition, the elevation of GFAP that occurs in CSF may prove useful as a biomarker of disease severity or progression in clinical studies of AxD. Indeed, Kyllerman et al. (2005) studied three patients, each of whom exhibited markedly elevated levels in their CSF compared with controls. A study of CSF levels of GFAP in a larger cohort of AxD patients is currently underway, along with an evaluation of blood levels. The validation of biomarkers that occur both in human patients as well as animal models will greatly facilitate the evaluation of candidate therapies in the future.

## Online data

Supplementary data

## References

[B1] Brenner M (1994). Structure and transcriptional regulation of the GFAP gene. Brain Pathol.

[B2] Brenner M, Goldman JE, Quinlan RA, Messing A, Parpura V., Haydon P. G. (2009). Alexander disease: a genetic disorder of astrocytes. Astrocytes in (Patho) Physiology of the Nervous System.

[B3] Brenner M, Johnson AB, Boespflug-Tanguy O, Rodriguez D, Goldman JE, Messing A (2001). Mutations in *GFAP*, encoding glial fibrillary acidic protein, are associated with Alexander disease. Nat Genet.

[B4] Chen H, Cabon F, Sun P, Parmantier E, Dupouey P, Jacque C, Zalc B (1993). Regional and developmental variations of GFAP and actin mRNA levels in the CNS of jimpy and shiverer mutant mice. J Mol Neurosci.

[B5] Chiu FC, Goldman JE (1984). Synthesis and turnover of cytoskeletal proteins in cultured astrocytes. J Neurochem.

[B6] Cho W, Brenner M, Peters N, Messing A (2010). Drug screening to identify suppressors of GFAP expression. Hum Mol Genet.

[B7] Cho W, Hagemann TL, Johnson DA, Johnson JA, Messing A (2009). Dual transgenic reporter mice as a tool for monitoring expression of GFAP. J Neurochem.

[B8] Cho W, Messing A (2009). Properties of astrocytes cultured from GFAP over-expressing and GFAP mutant mice. Exp Cell Res.

[B9] Cordeau P, Lalancette-Herbert M, Weng YC, Kriz J (2008). Live imaging of neuroinflammation reveals sex and estrogen effects on astrocyte response to ischemic injury. Stroke.

[B10] Cui C, Wani MA, Wight D, Kopchick J, Stambrook PJ (1994). Reporter genes in transgenic mice. Transgenic Res.

[B11] Cunningham R, Jany P, Messing A, Li L (2013). Protein changes in immunodepleted cerebrospinal fluid from transgenic mouse models of Alexander disease detected using mass spectrometry. J Proteome Res.

[B12] DeArmond SJ, Lee YL, Kretzschmar HA, Eng LF (1986). Turnover of glial filaments in mouse spinal cord. J Neurochem.

[B13] Delcourt N, Jouin P, Poncet J, Demey E, Mauger E, Bockaert J, Marin P, Galéotti N (2005). Difference in mass analysis using labeled lysines (DIMAL-K): a new, efficient proteomic quantification method applied to the analysis of astrocytic secretomes. Mol Cell Proteomics.

[B14] DeMattos RB, Bales KR, Parsadanian A, O’Dell DM, Foss EM, Paul SM, Holtzman DM (2002). Plaque-associated disruption of CSF and plasma amyloid-β (Aβ) equilibrium in a mouse model of Alzheimer's disease. J Neurochem.

[B15] Dowell JA, Johnson JA, Li L (2009). Identification of astrocyte secreted proteins with a combination of shotgun proteomics and bioinformatics. J Proteome Res.

[B16] Greco TM, Seeholzer SH, Mak A, Spruce L, Ischiropoulos H (2010). Quantitative mass spectrometry-based proteomics reveals the dynamic range of primary mouse astrocyte protein secretion. J Proteome Res.

[B17] Hagemann TL, Boelens W, Wawrousek E, Messing A (2009). Suppression of GFAP toxicity by αB-crystallin in mouse models of Alexander disease. Hum Mol Genet.

[B18] Hagemann TL, Connor JX, Messing A (2006). Alexander disease-associated glial fibrillary acidic protein mutations in mice induce Rosenthal fiber formation and a white matter stress response. J Neurosci.

[B19] Hagemann TL, Gaeta SA, Smith MA, Johnson DA, Johnson JA, Messing A (2005). Gene expression analysis in mice with elevated glial fibrillary acidic protein and Rosenthal fibers reveals a stress response followed by glial activation and neuronal dysfunction. Hum Mol Genet.

[B20] Kadurugamuwa JL, Modi K, Coquoz O, Rice B, Smith S, Contag PR, Purchio T (2005). Reduction of astrogliosis by early treatment of pneumococcal meningitis measured by simultaneous imaging, *in vivo*, of the pathogen and host response. Infect Immun.

[B21] Keene SD, Greco TM, Parastatidis I, Lee SH, Hughes EG, Balice-Gordon RJ, Speicher DW, Ischiropoulos H (2009). Mass spectrometric and computational analysis of cytokine-induced alterations in the astrocyte secretome. Proteomics.

[B22] Keller AF, Gravel M, Kriz J (2009). Live imaging of amyotrophic lateral sclerosis pathogenesis: disease onset is characterized by marked induction of GFAP in Schwann cells. Glia.

[B23] Krencik R, Weick JP, Liu Y, Zhang ZJ, Zhang SC (2011). Specification of transplantable astroglial subtypes from human pluripotent stem cells. Nat Biotechnol.

[B24] Kyllerman M, Rosengren L, Wiklund LM, Holmberg E (2005). Increased levels of GFAP in the cerebrospinal fluid in three subtypes of genetically confirmed Alexander disease. Neuropediatrics.

[B25] LaPash Daniels CM, Austin E, Rockney D, Jacka E, Hagemann TL, Johnson D, Johnson JA, Messing A (2012). Beneficial effects of Nrf2 overexpression in a mouse model of Alexander disease. J Neurosci.

[B26] Laping NJ, Teter B, Nichols NR, Rozovsky I, Finch CE (1994). Glial fibrillary acidic protein: regulation by hormones, cytokines, and growth factors. Brain Pathol.

[B27] Lein ES, Hawrylycz MJ, Ao N, Ayres M, Bensinger A, Bernard A, Boe AF, Boguski MS, Brockway KS, Byrnes EJ, Chen L, Chen L, Chen TM, Chin MC, Chong J, Crook BE, Czaplinska A, Dang CN, Datta S, Dee NR, Desaki AL, Desta T, Diep E, Dolbeare TA, Donelan MJ, Dong HW, Dougherty JG, Duncan BJ, Ebbert AJ, Eichele G, Estin LK, Faber C, Facer BA, Fields R, Fischer SR, Fliss TP, Frensley C, Gates SN, Glattfelder KJ, Halverson KR, Hart MR, Hohmann JG, Howell MP, Jeung DP, Johnson RA, Karr PT, Kawal R, Kidney JM, Knapik RH, Kuan CL, Lake JH, Laramee AR, Larsen KD, Lau C, Lemon TA, Liang AJ, Liu Y, Luong LT, Michaels J, Morgan JJ, Morgan RJ, Mortrud MT, Mosqueda NF, Ng LL, Ng R, Orta GJ, Overly CC, Pak TH, Parry SE, Pathak SD, Pearson OC, Puchalski RB, Riley ZL, Rockett HR, Rowland SA, Royall JJ, Ruiz MJ, Sarno NR, Schaffnit K, Shapovalova NV, Sivisay T, Slaughterbeck CR, Smith SC, Smith KA, Smith BI, Sodt AJ, Stewart NN, Stumpf KR, Sunkin SM, Sutram M, Tam A, Teemer CD, Thaller C, Thompson CL, Varnam LR, Visel A, Whitlock RM, Wohnoutka PE, Wolkey CK, Wong VY, Wood M, Yaylaoglu MB, Young RC, Youngstrom BL, Yuan XF, Zhang B, Zwingman TA, Jones AR (2007). Genome-wide atlas of gene expression in the adult mouse brain. Nature.

[B28] Levin-Allerhand J, McEwen BS, Lominska CE, Lubahn DB, Korach KS, Smith JD (2001). Brain region-specific up-regulation of mouse apolipoprotein E by pharmacological estrogen treatments. J Neurochem.

[B29] Liem RKH, Messing A (2009). Dysfunctions of neuronal and glial intermediate filaments in disease. J Clin Invest.

[B30] Luo J, Ho P, Steinman L, Wyss-Coray T (2008). Bioluminescence *in vivo* imaging of autoimmune encephalomyelitis predicts disease. J Neuroinflammation.

[B31] Martin PM, O’Callaghan JP (1995). A direct comparison of GFAP immunocytochemistry and GFAP concentration in various regions of ethanol-fixed rat and mouse brain. J Neurosci Methods.

[B32] McAsey ME, Cady C, Jackson LM, Li M, Randall S, Nathan BP, Struble RG (2006). Time course of response to estradiol replacement in ovariectomized mice: brain apolipoprotein E and synaptophysin transiently increase and glial fibrillary acidic protein is suppressed. Exp Neurol.

[B33] McCall MA, Gregg RG, Behringer RR, Brenner M, Delaney CL, Galbreath EJ, Zhang CL, Pearce RA, Chiu SY, Messing A (1996). Targeted deletion in astrocyte intermediate filament (*Gfap*) alters neuronal physiology. Proc Natl Acad Sci USA.

[B34] Messing A, Brenner M, Kettenmann H., Ransom B. (2013). Genetic disorders affecting astrocytes. Neuroglia.

[B35] Messing A, Brenner M, Feany MB, Nedergaard M, Goldman JE (2012). Alexander disease. J Neurosci.

[B36] Messing A, Daniels CM, Hagemann TL (2010). Strategies for treatment in Alexander disease. Neurotherapeutics.

[B37] Messing A, Head MW, Galles K, Galbreath EJ, Goldman JE, Brenner M (1998). Fatal encephalopathy with astrocyte inclusions in GFAP transgenic mice. Am J Pathol.

[B38] Mignot C, Delarasse C, Escaich S, della Gaspera B, Noé E, Colucci-Guyon E, Babinet C, Pekny M, Vicart P, Boespflug-Tanguy O, Dautigny A, Rodriguez D, Pham-Dinh D (2007). Dynamics of mutated GFAP aggregates revealed by real-time imaging of an astrocyte model of Alexander disease. Exp Cell Res.

[B39] Moore NH, Costa LG, Shaffer SA, Goodlett DR, Guizzetti M (2009). Shotgun proteomics implicates extracellular matrix proteins and protease systems in neuronal development induced by astrocyte cholinergic stimulation. J Neurochem.

[B40] Mor-Vaknin N, Punturieri A, Sitwala K, Markovitz DM (2003). Vimentin is secreted by activated macrophages. Nat Cell Biol.

[B41] Morrison RS, de Vellis J, Lee YL, Bradshaw RA, Eng LF (1985). Hormones and growth factors induce the synthesis of glial fibrillary acidic protein in rat brain astrocytes. J Neurosci Res.

[B42] Palfreyman JW, Thomas DG, Ratcliffe JG, Graham DI (1979). Glial fibrillary acidic protein (GFAP): purification from human fibrillary astrocytoma, development and validation of a radioimmunoassay for GFAP-like immunoactivity. J Neurol Sci.

[B43] Petzold A, Keir G, Green AJE, Giovannoni G, Thompson EJ (2004). An ELISA for glial fibrillary acidic protein. J Immunol Methods.

[B44] Price JC, Guan S, Burlingame A, Prusiner SB, Ghaemmaghami S (2010). Analysis of proteome dynamics in the mouse brain. Proc Natl Acad Sci USA.

[B45] Prust M, Wang J, Morizono H, Messing A, Brenner M, Gordon E, Hartka T, Sokohl A, Schiffmann R, Gordish-Dressman H, Albin R, Amartino H, Brockman K, Dinopoulos A, Dotti MT, Fain D, Fernandez R, Ferreira J, Fleming J, Gill D, Griebel M, Heilstedt H, Kaplan P, Lewis D, Nakagawa M, Pedersen R, Reddy A, Sawaishi Y, Schneider M, Sherr E, Takiyama Y, Wakabayashi K, Gorospe JR, Vanderver A (2011). *GFAP* mutations, age of onset, and clinical sub-types in Alexander disease. Neurology.

[B46] Stone DJ, Song Y, Anderson CP, Krohn KK, Finch CE, Rozovsky I (1998). Bidirectional transcription regulation of glial fibrillary acidic protein by estradiol *in vivo* and *in vitro*. Endocrinology.

[B47] Tamgüney G, Francis KP, Giles K, Lemus A, DeArmond SJ, Prusiner SB (2009). Measuring prions by bioluminescence imaging. Proc Natl Acad Sci USA.

[B48] Tanaka KF, Takebayashi H, Yamazaki Y, Ono K, Naruse M, Iwasato T, Itohara S, Kato H, Ikenaka K (2007). The murine model of Alexander disease: analysis of GFAP aggregate formation and its pathological significance. Glia.

[B49] Tang G, Xu Z, Goldman JE (2006). Synergistic effects of the SAPK/JNK and the proteasome pathway on glial fibrillary acidic protein (GFAP) accumulation in Alexander disease. J Biol Chem.

[B50] Tang G, Yue Z, Talloczy Z, Hagemann T, Cho W, Messing A, Sulzer DL, Goldman JE (2008). Autophagy induced by Alexander disease-mutant GFAP accumulation is regulated by p38/MAPK and mTOR signaling pathways. Hum Mol Genet.

[B51] Watts JC, Gilesa BK, Grilloa SK, Lemusc A, DeArmond SJ, Prusiner SB (2011). Bioluminescence imaging of Aβ deposition in bigenic mouse models of Alzheimer's disease. Proc Natl Acad Sci USA.

[B52] Westgard JO (2008). Basic Method Validation.

[B53] Zhu LY, Ramboz S, Hewitt D, Boring L, Grass DS, Purchio AF (2004). Non-invasive imaging of GFAP expression after neuronal damage in mice. Neurosci Lett.

