# Supplementary material for: GFAP expression as an indicator of disease severity in mouse models of Alexander disease
Source: ASN Neuro. 2013 Mar 21;5(2):e00109. doi: 10.1042/AN20130003 (PMC3604736; doi:10.1042/AN20130003)
Supplement: Supplementary data [file an005e109add.pdf]

ASN NEURO RESEARCH ARTICLE

ASN NEURO 5(2):art:e00109.doi:10.1042/AN20130003

OPEN ACCESS

GFAP expression as an indicator of disease severity in mouse models of Alexander disease

Paige L. Jany*, Tracy L. Hagemann† and Albee Messing‡[1]
*Cellular and Molecular Pathology Training Program, University of Wisconsin-Madison, WI, U.S.A.
†Waisman Center, University of Wisconsin-Madison, WI, U.S.A.
‡Waisman Center and Department of Comparative Biosciences, University of Wisconsin-Madison, WI, U.S.A.

SUPPLEMENTARY DATA

Supplementary Figure S1 is on the following page

[1]To whom correspondence should be addressed (email messing@waisman.wisc.edu).
© 2013 The Author(s) This is an Open Access article distributed under the terms of the Creative Commons Attribution Licence (CC-BY) (http://creativecommons.org/licenses/by/3.0/) which permits unrestricted use, distribution and reproduction in any medium, provided the original work is properly cited.

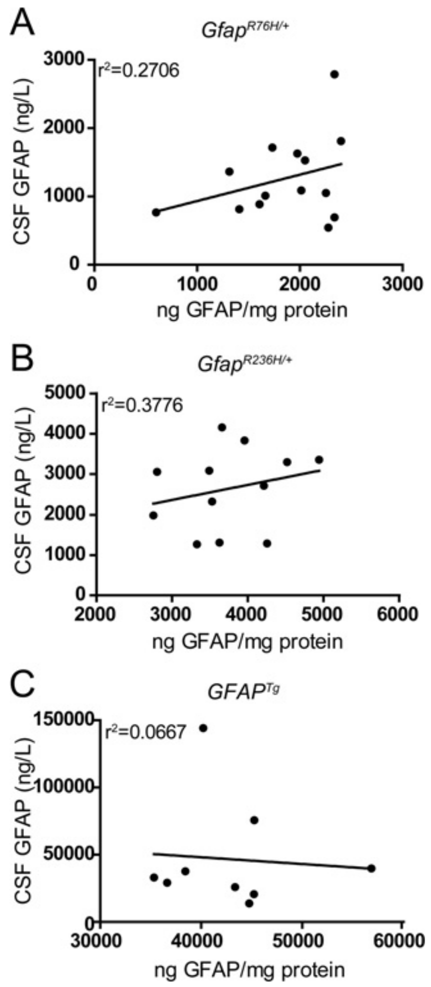

Figure S1 Correlation between GFAP levels in CSF and GFAP levels in the brain
Within each of the AxD mouse model groups, there was no correlation between the concentration of GFAP in CSF (ng/l GFAP) and in the brain (ng GFAP/mg protein) in individual mice. (**A**) *Gfap*$^{R76H/+}$ ($n = 14$), (**B**) *Gfap*$^{R236H/+}$ ($n = 12$) or (**C**) *GFAP*^{Tg} ($n = 9$). Each data point represents one mouse.

© 2013 The Author(s) This is an Open Access article distributed under the terms of the Creative Commons Attribution Licence (CC-BY) (http://creativecommons.org/licenses/by/3.0/) which permits unrestricted use, distribution and reproduction in any medium, provided the original work is properly cited.
